# Dilated Cardiomyopathy with Increased SR Ca^2+^ Loading Preceded by a Hypercontractile State and Diastolic Failure in the α_1C_TG Mouse

**DOI:** 10.1371/journal.pone.0004133

**Published:** 2009-01-06

**Authors:** Su Wang, Bruce Ziman, Ilona Bodi, Marta Rubio, Ying-Ying Zhou, Karen D'Souza, Nanette H. Bishopric, Arnold Schwartz, Edward G. Lakatta

**Affiliations:** 1 Laboratory of Cardiovascular Science, Gerontology Research Center, National Institute on Aging, Baltimore, Maryland, United States of America; 2 Institute of Molecular Pharmacology and Biophysics, Department of Surgery, University of Cincinnati, College of Medicine, Cincinnati, Ohio, United States of America; 3 Department of Molecular and Cellular Pharmacology, Medicine and Pediatrics, University of Miami Miller School of Medicine, Miami, Florida, United States of America; Leiden University Medical Center, Netherlands

## Abstract

Mice over-expressing the α_1−_subunit (pore) of the L-type Ca^2+^ channel (α_1C_TG) by 4months (mo) of age exhibit an enlarged heart, hypertrophied myocytes, increased Ca^2+^ current and Ca^2+^ transient amplitude, but a normal SR Ca^2+^ load. With advancing age (8–11 mo), some mice demonstrate advanced hypertrophy but are not in congestive heart failure (NFTG), while others evolve to frank dilated congestive heart failure (FTG). We demonstrate that older NFTG myocytes exhibit a hypercontractile state over a wide range of stimulation frequencies, but maintain a normal SR Ca^2+^ load compared to age matched non-transgenic (NTG) myocytes. However, at high stimulation rates (2–4 Hz) signs of diastolic contractile failure appear in NFTG cells. The evolution of frank congestive failure in FTG is accompanied by a further increase in heart mass and myocyte size, and phospholamban and ryanodine receptor protein levels and phosphorylation become reduced. In FTG, the SR Ca^2+^ load increases and Ca^2+^ release following excitation, increases further. An enhanced NCX function in FTG, as reflected by an accelerated relaxation of the caffeine-induced Ca^2+^ transient, is insufficient to maintain a normal diastolic Ca^2+^ during high rates of stimulation. Although a high SR Ca^2+^ release following excitation is maintained, the hypercontractile state is not maintained at high rates of stimulation, and signs of both systolic and diastolic contractile failure appear. Thus, the dilated cardiomyopathy that evolves in this mouse model exhibits signs of both systolic and diastolic failure, but not a deficient SR Ca^2+^ loading or release, as occurs in some other cardiomyopathic models.

## Introduction

Much evidence has accumulated since the first cytosolic Ca^2+^ transient measurements in cardiac muscle from heart failure (HF) patients [1] to support a role for alterations in myocyte “Ca^2+^ handling” in the pathophysiology of HF. Although multiple alterations in various aspects of myocyte excitation-contraction coupling have been observed in HF, the central mechanism of the reduced Ca^2+^ transient amplitude has been emphasized and has been attributed to a reduction of sarcoplasmic reticulum (SR) Ca^2+^ content [1]–[7].

Longitudinal assessment of cardiac structure and function in a novel transgenic mouse that over expresses the α_1C_-subunit of the L-type Ca^2+^ channel (α_1C_TG) indicates that like droves of other “boutique mice” (**Table S1**), this mouse, develops cardiac hypertrophy, during which adaptive Ca^2+^ regulatory mechanisms are mobilized. The remarkable orchestration among cardiomyocyte Ca^2+^ regulatory proteins in this model at 4months (mo) is instructive because it provides clues with respect to coordinated, adaptive remodeling of Ca^2+^ regulation to maintain a normal SR Ca^2+^ load at 4 mo of age [4]. Specifically, during the hypertrophic, pre-HF stage, while an L-type current of a larger amplitude triggers larger Ca^2+^ release from the SR to produce a whole cell Ca^2+^ transient of increased amplitude, neither the SR Ca^2+^ load, as assessed by caffeine induced Ca^2+^ release, nor diastolic cytosolic Ca^2+^ levels, nor Ca^2+^ spark characteristics, are altered [4]. An overexpression of NCX protein, which enhances Ca^2+^ efflux to balance the enhanced Ca^2+^ influx via the overexpressed L-type Ca^2+^ channel prevents excess cytosolic calcium loading [4].

Between 8–11 mo of age, however, the adapted, hypertrophic heart of the α_1C_TG mouse maladapts into a dilated, lethal cardiomyopathy [4]. The hypothesis of the present study is that adaptations in Ca^2+^ regulation observed at a younger age (4 mo) in NTG cells wane with the evolution of more advanced hypertrophy and HF that accompany advancing age. Specifically, we hypothesized that these adaptations become compromised in the non-failing advanced hypertrophic stage (NFTG), and “fail” outright as the end-stage dilated congestive failure (FTG) evolves.

## Results

### Heart and Myocyte Size

**Figure 1:**
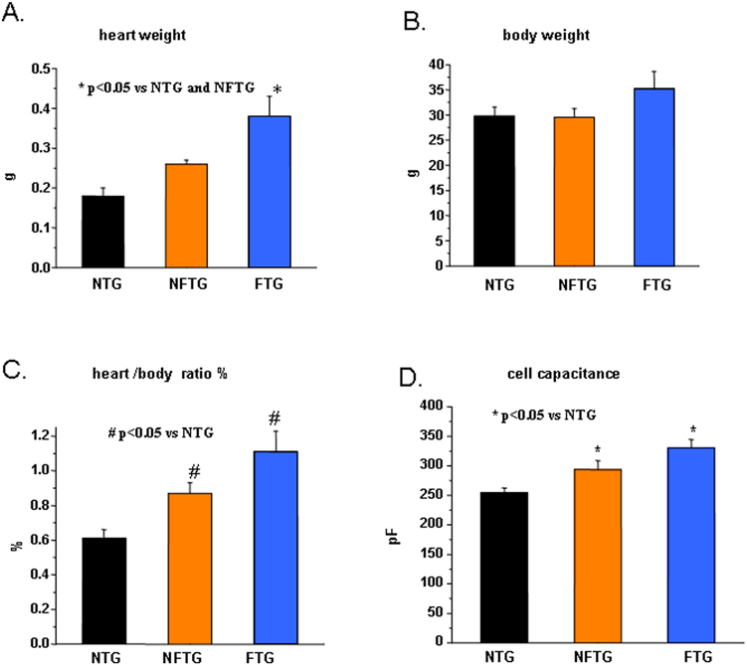
Development of cardiac hypertrophy and heart failure in Ca_v_1.2α_1C_ transgenic mice. (A) Measurement of heart weight, (B) body weight, (C) heart/body weight ratio and (D) size of myocytes estimated from cell capacitance in Non-Transgenic (NTG), Non-Failing Ca_v_1.2α_1C_-Transgenic (NFTG) and Failing Ca_v_1.2α_1C_-Transgenic (FTG) mouse groups (8–12 mo). n =  9 NTG, 4 NFTG and 4 FTG in (A,B and C) and 26 NTG, 12 NFTG and 31 FTG in (D).

Heart weight in FTG was over two-fold greater than in NTG (Figure 1A). Body weight did not differ between NTG and NFTG but body weight increased by 15% in FTG, likely as a result of fluid accumulation (Figure 1B). The heart weight/body weight increased by 43% in NFTG vs NTG, by 27% in FTG vs. NFTG, and by 82% in FTG vs NTG (Figure 1C). The average myocyte size, estimated from cell capacitance, reflected the relative heart mass. It increased by 25% in NFTG vs NTG, and in FTG further increased by 29%. Thus, myocyte size in failing FTG averaged 61% more than that in NTG (Figure 1D).

### Ca^2+^ Current and Electrical Stimulation-Induced Ca^2+^ Transients

**Figure 2:**
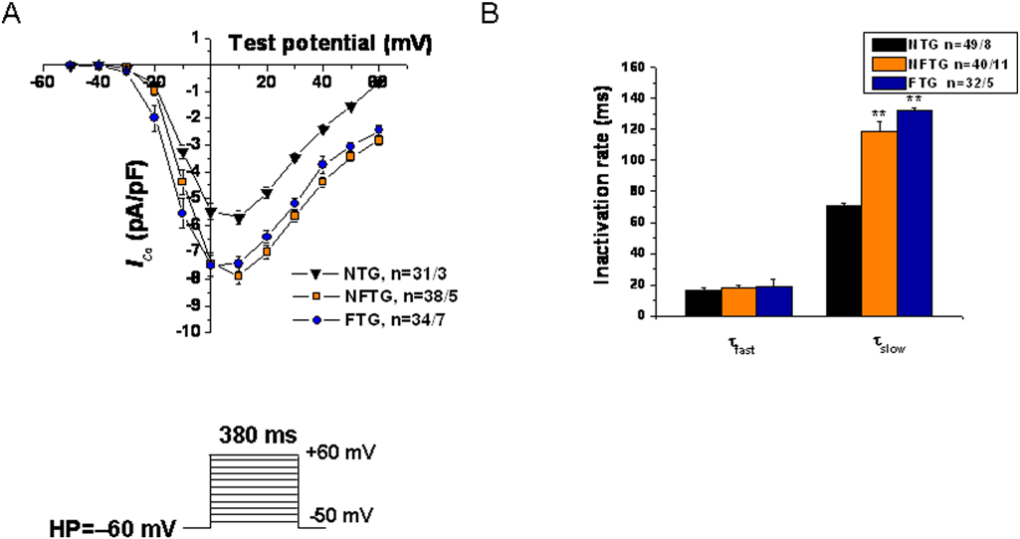
Electrophysiological effects of Ca_v_1.2α_1C_ overexpression in mouse ventricular myocytes. (A) Averaged peak current-voltage relationships demonstrate significant increase of *I*_Ca,L_ density at multiple depolarizing pulses in NFTG and FTG compared with NTG cardiomyocytes. The voltage protocol used to record *I*_Ca,L_ is shown in the inset. (B) Inactivation time constants (τ_fast_ and τ_slow_) were determined from *I*_Ca,L_ traces depolarized to +10 mV fitted by double exponential equation: Y = Y_min_+ A_1_×[1−exp(−t/τ_fast_)] + A_2_×[1−exp(−t/τ_slow_)], where Y is the fraction of recovery, A_1_ and A_2_ are the maximum values of the fast and slow component, and τ_fast_ and τ_slow_ are the time constants, respectively.

**Figure 3:**
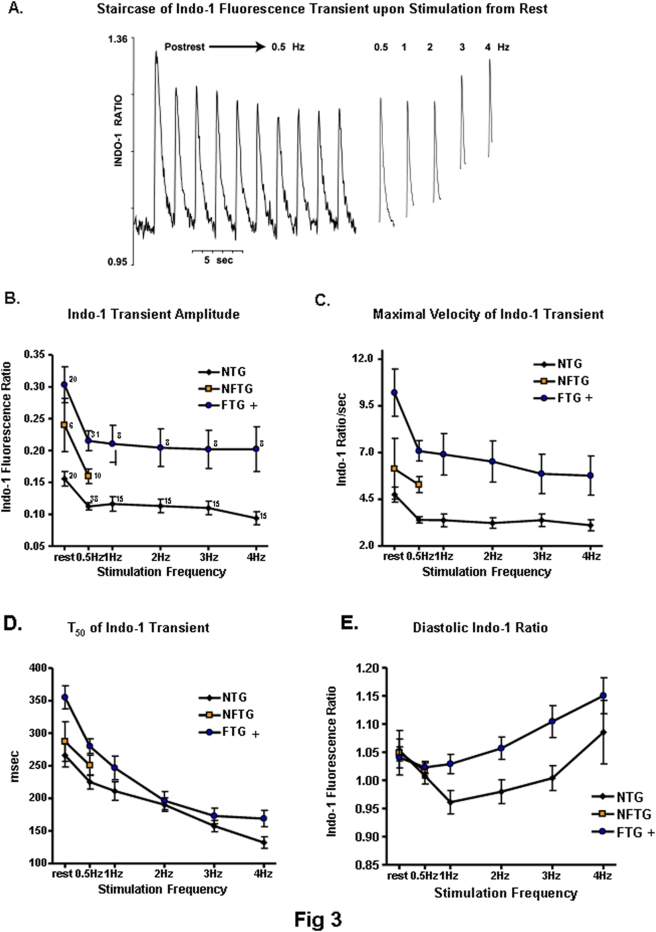
Changes in Ca^2+^ transient and decline in response to an increase in frequency. (A) Original representative recording showing Indo-1 fluorescence (410/490) transients in response to electrical stimulation following a two-minute rest and during steady state stimulation at 0.5 to 4 Hz in FTG. (B) Calcium transient amplitude, (C) maximal velocity of Ca^2+^ transient upstroke, (D) time to 50% decay (T_50_) and (E) diastolic ratio in Indo-1 loaded mouse ventricular cardiomyocytes. + ANOVA across all stimulation rates *P<0.01* vs NTG

Peak *I*_Ca_ density in NFTG and FTG was increased compared to NTG, despite a significant increase in cell capacitance (293.5±15.02 pF, 330.93±13.53 pF vs 254.61±7.76 pF, respectively). No statistically significant difference was observed between NFTG and FTG (Figure 2A). Inactivation rate constants of *I*_CaL_ are illustrated in Figure 2B. Tau fast did not differ among groups but Tau slow was prolonged in both TG groups compared to NTG. In rodent myocytes, the rested state Ca^2+^ transient in Figure 3 is usually the maximal that can be achieved, due to a maximum SR Ca^2+^ load and release achieved during rest. Upon continued stimulation from rest a typical negative staircase in Ca^2+^ transient typically occurs. This reflects, in part, a net reduction in cell and SR Ca^2+^ loading.

The amplitude of the Indo-1 fluorescence transient (peak-rest or peak-diastolic level) elicited by electrical stimulation declined sharply between the first stimulation following rest and steady state stimulation at 0.5 Hz. There was little further decline thereafter as the stimulation rate was increased (Figure 3B). Following rest, and at all frequencies of stimulation, the Indo-1 transient amplitude in FTG was greater than in NTG. In NFTG group, data were available only at rest and 0.5 Hz. The Indo 1 fluorescent transient at these two frequencies was intermediate between that of FTG and NTG.

The maximum rate of rise (V_MAX_) of the Indo fluorescent transient (Figure 3C) reports the amplitude of the SR Ca^2+^ release flux with fidelity [1], [8], [9]. Differences among the three groups in V_MAX_ upon stimulation from rest were similar to those of the amplitude of the fluorescence transient. This suggests that the decline in the Ca^2+^ transient amplitude from the post-rest state reflects a decrease in excitation induced SR Ca^2+^ release. A greater Ca^2+^ transient V_MAX_ across the range of stimulation rates in FTG indicates that the magnitude of excitation-induced SR release in FTG is greater than in NTG.

Continued stimulation from rest accelerated the decay of the Ca^2+^ transient (Figure 3D). The T_50_ of relaxation was prolonged in FTG vs. NTG across the range of stimulation; the difference is more marked in the resting state beat than that of 0.5 Hz. The T_50_ measured over the limited range of frequencies in NFTG did not differ from NTG or FTG.

At rest there was no difference in the resting Indo-1 fluorescence ratio among the three groups of cells (Figure 3E). Upon regular electrical stimulation from rest the diastolic Indo fluorescence at rates up to 4 Hz in NTG did not exceed that at rest. In contrast, in FTG the diastolic fluorescence increases as the stimulation rate increases, exceeding that at rest by 10% at 4 Hz. Note that the difference in the resting fluorescence ratio prior to stimulation and diastolic fluorescence ratio at 4 Hz was about 0.10 (Figure 3E). The amplitude of fluorescence transient elicited by excitation at 4 Hz was about 0.22 (Figure 3B). Thus, even if only 50% of the change from the resting Indo fluorescence reports a true change in diastolic cytosolic Ca^2+^, the increase in diastolic Ca^2+^ in FTG with stimulation is substantial. During excitation at 4 Hz, this increase averaged 20–25% of the Ca^2+^ transient amplitude.

### Caffeine-Induced Ca^2+^ Transients

**Figure 4:**
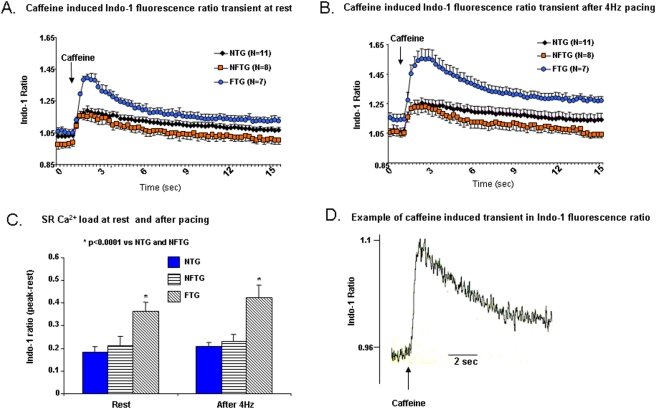
Effect of caffeine on Ca^2+^ transients. (A) Represents an average of the tracings of caffeine-induced Ca^2+^ transients (20 mmol/L) prior to and (B) after stimulation at 4 Hz and Indo-l loaded isolated cardiomyocytes from all three groups. (C) Comparison of average peak values of caffeine-induced Ca^2+^ transients or SR calcium load at rest and after 4 Hz stimulation. SR calcium load was significantly increased under both protocols in FTG group. (D) Representative example of rapid application of caffeine to a cardiomyocyte.

We assessed the SR Ca^2+^ load by a rapid, brief application of caffeine to the cell. In a prior study, no difference was noted, in the amplitude of caffeine-induced Indo fluorescent transient between NTG and α_1C_TG at 4 mo and 9 mo. Likewise, in the present study, there was no difference in the amplitude of the caffeine-induced transient between NFTG and NTG (Figure 4). But the caffeine-induced transient in FTG was increased compared to NFTG or NTG groups (Figure 4). Note that the amplitude of the caffeine transient at 4 Hz did not vary from that at rest, in contrast to the amplitude of the transient induced by electrical stimulation, which is much larger at rest than that at 4 Hz (Figure 4A). Thus, by rough calculations, the SR “fractional release” in response to electrical stimulation, *i.e.*, the ratio of the electrical stimulation and caffeine Ca^2+^ transients' amplitudes was reduced at 4 Hz compared to rest. The extent of this reduction was comparable in NTG and FTG: at rest it averaged 0.85 and 0.83 respectively, and at 4 Hz was 0.39 and 0.47, respectively.

**Figure 5:**
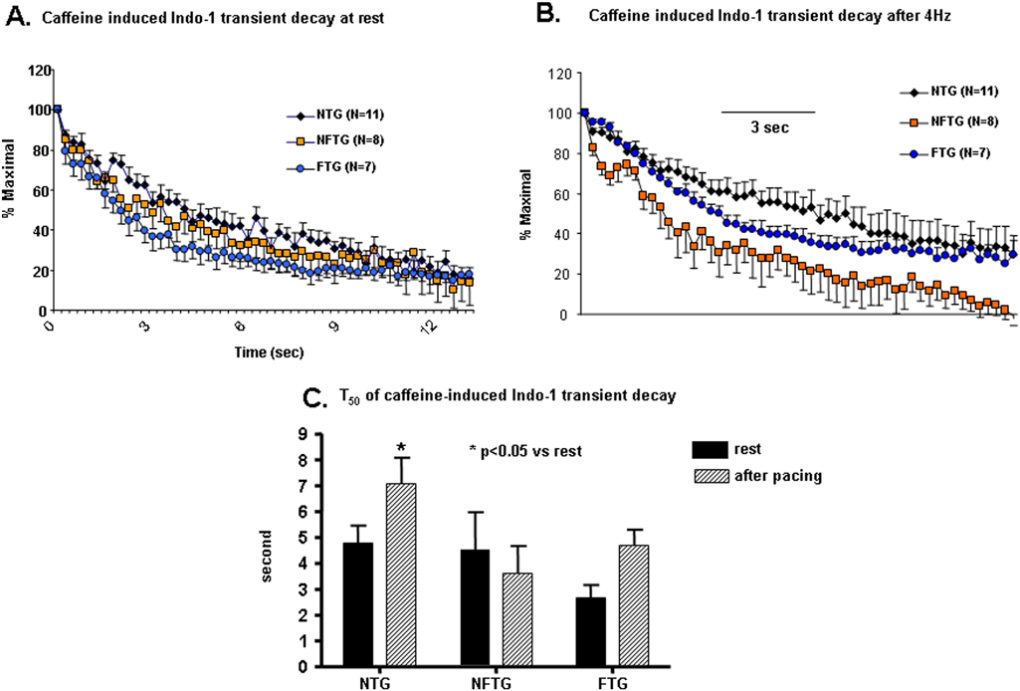
Changes in Ca^2+^ transient kinetics in response to caffeine application. (A) Average caffeine-induced Indo-1 fluorescence Ca^2+^ transient ratio (410/490), normalized to their peak amplitude at rest and (B) after stimulation at 4 Hz in all three groups. (C) Average T_50_ of the normalized caffeine-induced Indo-1 fluorescent Ca^2+^ transients in rest and after stimulation at 4 Hz.

Figure 4A, B also shows that the decay rate of the caffeine transient is accelerated in FTG. This is more clearly illustrated by normalization of the data during the decay to the peak amplitude in each cell (Figure 5A and B). On an average, the T_50_ of relaxation of the caffeine-induced Indo transient was accelerated by 50% in FTG vs. NTG (Figure 5C). In NFTG, the T_50_ did not differ from that of NTG following rest. During continuous pacing, however, the T_50_ in NFTG became less than NTG. The decay of the caffeine transient following 4 Hz stimulation was slower than at rest in both NTG and FTG. But FTG T_50_ still remained substantially accelerated compared to NTG. NFTG showed no increase or difference from FTG (Figure 5C). The ability of NFTG to maintain the same T_50_ of the caffeine transient following 4 Hz as at rest suggests that NCX reserve may be greater in NFTG than in FTG or NTG cells.

A comparison of Figure 5 and Figure 3D reveals that the group differences between FTG and NTG in the T_50_ of the caffeine induced transient decay and the T_50_ of electrical stimulation induced transient are reversed: the electrical stimulation induced transient T_50_, which largely reflects SR Ca^2+^ pumping, was more rapid in NTG than FTG. The caffeine induced transient T_50_, reflecting mostly NCX Ca^2+^ extrusion, was more rapid in FTG than NTG. However, a relative failure of SERCA2a and NCX to dissipate cytosolic Ca^2+^ at high rates of electrical stimulation in FTG likely explains the failure to regulate the diastolic Indo fluorescence (Figure 3E).

### Contraction Characteristics

Changes in Ca^2+^ release into and removal from the cytosol, as observed in FTG, may result in contractile impairment. We first determined the extent to which Indo-1 loading affects myocyte contractile properties in both FTG and NTG cells. The contractile characteristics, i.e., twitch amplitude, shortening velocity, and relaxation time were severely distorted by Indo-1 in both groups of cells; the buffering effect on twitch amplitude was greater in FTG than in NTG myocytes (**Table S2**). To assess artifact-free contraction characteristics, we performed additional studies in non-Indo-1 loaded cells to analyze Ca^2+^ buffering.

**Figure 6:**
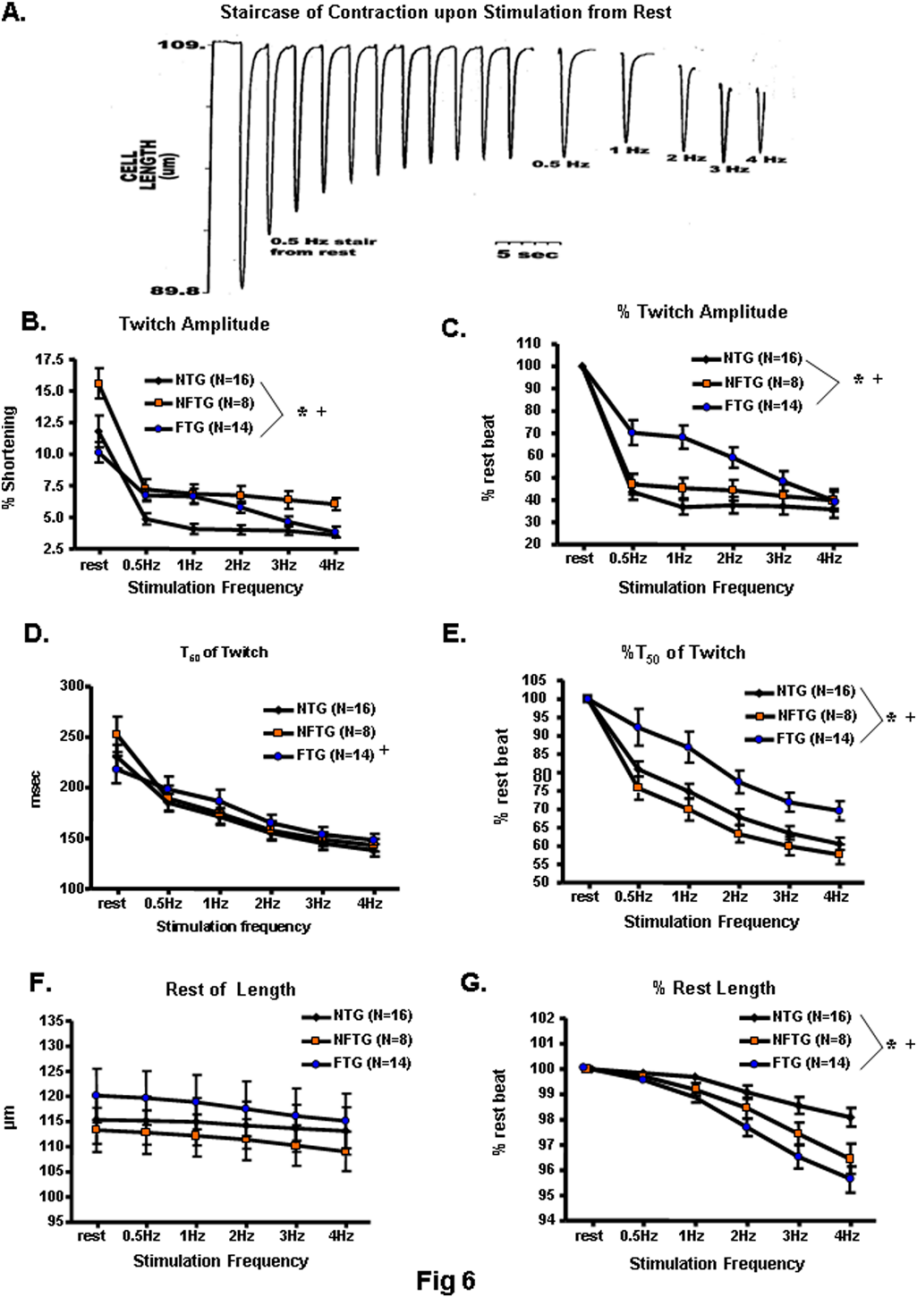
Contractile parameters of isolated ventricular myocytes from NTG, NFTG and FTG hearts. (A) Representative example of the negative staircase of twitch contractions following rest during stimulation at 0.5 Hz and during steady state stimulation at 0.5–4 Hz in FTG. (B–G) Average contraction characteristics in response to electrical stimulation following rest and during steady state stimulation at varying frequencies. (B) Figure illustrates absolute data in non-Indo-1 AM-loaded, isolated cardiomyocytes, (G,F) are expressed as percentage change from 0.5 Hz values in (C,E and G). (B,C) Show contraction amplitude (or Twitch Amplitude). (D,E) Illustrate changes in the maximal rate of contraction. (F,G) Depict diastolic cardiomyocyte length. * All 3 groups differ from each other, ANOVA *P<0.03*; + FTG interaction with NTG and NFTG, ANOVA *P<0.01*.

In response to the initial excitation following rest the absolute contraction amplitude was greater in the NFTG than in FTG or NTG (Figure 6A–C). As stimulation from rest continues, the normal response is for contraction amplitude to decrease, as it does in NTG. During stimulation at low rates (0.5–1 Hz), the relative decline in NTG was greater than that in FTG or NFTG: the absolute contraction amplitude in NFTG and FTG exceeds that in NTG by 50%. This is consistent with a hypercontractile stage in the evolution of cardiomyopathy in other models, including humans [9]. Greater contraction amplitude in TG cells suggests a relative inability to appropriately reduce the cell Ca^2+^ load in this context [1], [10], [11]. Contraction amplitude remained higher in NFTG cells than in NTG cells during steady state stimulation at 2–4 Hz, but in FTG it dropped to that of NTG. Note that failure of FTG to maintain augmented contraction amplitude vs. NTG, or one equivalent to NFTG at the higher stimulation rates, occurred in the context of a larger SR Ca^2+^ release flux and Ca^2+^ transient amplitude in FTG vs. NTG (Figure 4).

The absolute T_50_ of cell relengthening (relaxation), like that of the Indo fluorescence transient (Figure 3), was prolonged in FTG in the initial excitation following rest. But T_50_ of relaxation converged toward other groups with increasing stimulation rates (Figure 6D, E). The absolute cell length at rest remained constant among the 3 groups (Figure 6F). Both transgenic groups showed an inability to maintain a normal diastolic length as stimulation frequency increased (Figure 6G). In FTG, this could result from an inability to maintain a normal diastolic Ca^2+^, reflected by failure to maintain a normal diastolic Indo-1 fluorescence ratio (Figure 3E). Thus, in myocytes from the NFTG hearts, electrical stimulation at high rates uncovered a diastolic contractile failure: in the myocytes from the grossly dilated hearts of congested FTG mice, high rates of stimulation provoked both systolic contractile failure and severe diastolic contractile failure. A “relative contractile, i.e., failure” also occurred within FTG at high stimulation rates, as the reduction in contraction amplitude was not accompanied by a reduction in Ca^2+^ release following excitation (Figure 4B vs. 6B). This could, in part, be due to a failure to maintain diastolic Ca^2+^ levels, but cannot be attributed to a failure to release Ca^2+^ into the cytosol following excitation.

### Expression of Ca^2+^ cycling proteins

**Figure 7:**
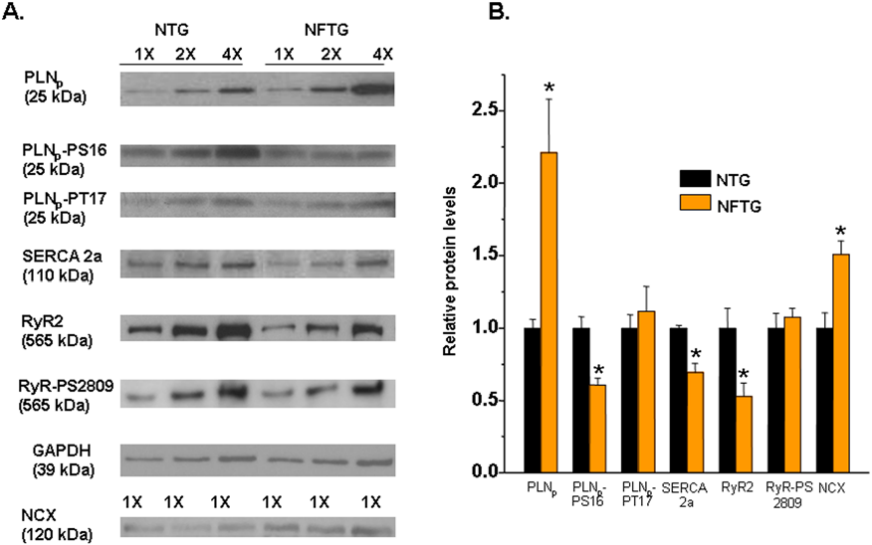
A comparison of Ca^2+^ cycling protein levels in NTG vs. NFTG measured by Western blot analysis. (A) Representative Western blots in NTG and NFTG hearts. (B) Column diagram of average protein levels. All samples were done in duplicates; n = 4–8; *P<0.05 by Student's *t*-test.

Quantitative Western Blot techniques comparing NFTG and NTG (8–11 mo) showed: a 30% decrease in SERCA2a levels; 50% increase in NCX; 120% increase in PLN_p_ (PLN pentamer) and 73% and 54% decrease in its phosphorylation levels of PS16 and PT17, respectively normalized to total PLN_p_; by at 48% decrease in RyR2 levels but 115% hyperphosphorylation of RyRs at Ser2089 (Figure 7).

**Figure 8:**
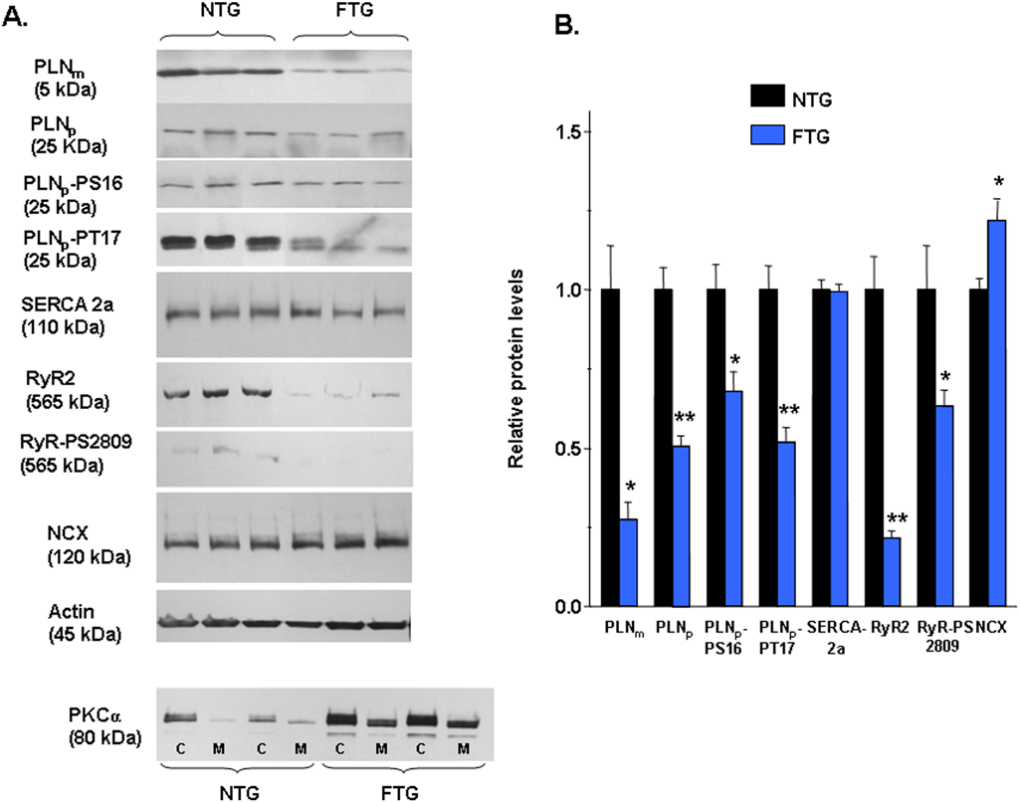
Western blot analysis of Ca^2+^ regulatory proteins in FTG and NTG hearts. (A) Representative Western blots of Ca^2+^ regulatory proteins from NTG and FTG. (B) Analysis of calcium regulatory protein abundance normalized to actin. All samples were done in duplicates; n = 4–6; *P<0.05 and **P<0.001 by Student's *t*-test.

Western blot analysis comparing FTG and NTG (8–11 mo) showed: an 80% decrease in the PLN monomer (PLN_m_); a 57% decrease in the PLN_p_; a 36% decrease in PLN-PS16; a 60% decrease in PLN_p_-PT17; a 5% decrease in SERCA2a; a 20% increase in NCX; a 43% increase in PKCα; and surprisingly, a 70% decrease in RyR2 and 25% decrease in RyR2-PS2809 (Figure 8).

## Discussion

There are several novel results of the present study. In NFTG at 8–11 mo of age, heart mass and average myocyte sizes are increased by about 25% relative to age-matched NTG as is the case in 4 mo α_1C_TG vs. NTG cells [4]. During the evolution from NFTG to the FTG state, both heart mass and myocyte size are increased markedly. At low rates of stimulation several other features of 4 mo α_1C_TG myocytes, [4] including an increased [Ca^2+^]_i_ transient elicited by electrical stimulation and a normal SR Ca^2+^ load, are retained in 9 mo NFTG. Cardiomyocytes from both the NFTG and FTG myocytes at 8–11 mo showed a marked increase in *I*_Ca,L_ amplitude compared to NTG. In FTG, in contrast to NFTG, both, the Ca^2+^ transient amplitude following excitation and the SR Ca^2+^ load are increased. While systolic Ca^2+^ release in FTG is higher than in NFTG, and continues to be elevated during stimulation over a wide range of rates, mechanisms that regulate Ca^2+^ removal from the cytosol are further impaired, and diastolic [Ca^2+^]_i_ levels increase excessively during stimulation at high frequencies, causing a more marked impairment of diastolic myocyte length than observed in NFTG (see below). Thus, although NCX function, as reflected in a reduced T_50_ of the caffeine transient relaxation, is enhanced in FTG, this is not sufficient to maintain a normal diastolic Ca^2+^ during high rates of stimulation.

Contractile characteristics of α_1C_TG myocytes have not been previously measured. Over a wide range of stimulation rates, the NFTG exhibit a hypercontractile state accompanied by diastolic failure due to an inability to maintain normal diastolic length. But in FTG, despite increased Ca^2+^ transient amplitude, the contraction amplitude across a broad range of stimulation rates FTG is not greater than in NFTG. This indicates that a relative systolic contractile failure has occurred with evolution from the NFTG to FTG state. At high rates of stimulation, the contraction amplitude of FTG declines below that of NFTG to the level in NTG without a decline in SR Ca^2+^ release and is accompanied by marked diastolic abnormalities. Thus NFTG and FTG differ in that the latter exhibit frank contractile systolic failure and more severe Ca^2+^ and contractile diastolic failure than do the former.

Diastolic abnormalities accompany hypercontractile function in other animal experimental models [1]. The hypertrophic SHR myocytes studied at 23°C present a markedly similar profile to NFTG described herein [1], [4], [12]. In contrast, myocytes from some other models of cardiac hypertrophy exhibit both reduced Ca^2+^ transient and contraction amplitudes. A common clinical correlate of NFTG may be hypertrophic non-dilated cardiomyopathy [13].

A hypercontractile state is attributable, at least in part, to an increased cytosolic [Ca^2+^]_i_ transient following excitation and may involve myofilaments, Ca^2+^ energetic mechanisms, matrix properties or interactions of all.

It has recently been suggested that, like NFTG myocytes in the present study, an abnormal frequency-dependent response of human heart failure is a pathophysiologic signature of the cardiomyopathic state [1], [2]. Both the hypercontractile state of NFTG myocytes, like the failing human heart, may reflect an altered functional balance between Ca^2+^ influx, reuptake and extrusion from the cell [11] and can occur in the absence of systolic failure [14]. In failing ventricular myocardium, the positive FFR disappears and becomes flat or negative [1], [2]. Multiple alterations in Ca^2+^ handling determine negative staircase in a cellular heart failure model [15]. Interestingly, in the MLP knockout mouse with dilated cardiomyopathy (enhanced basal SERCA activity) increased PLN phosphorylation is believed to limit FFR [16]. In human heart failure reduced expression of SERCA has been demonstrated [1], [2] however in animal studies, there is less consistency [1]. PLN expression levels are, however insufficient to evaluate the regulation of SERCA, as the level of inhibition of SERCA by PLN depends on the degree of phosphorylation. A decrease in the level of PLN phosphorylation at Ser16 (mainly affected by PKA activity) has been reported in some papers in heart failure, as against decreased levels of Thr17 phosphorylation (affected by the decreased CaMKII activity)[2].

Our results show that the maintenance of a normal myocyte SR Ca^2+^ load afforded to older NFTG mice fails as the dilated, congested cardiomyopathic state evolves. In marked contrast to most other cardiomyopathies, the excitation-induced Ca^2+^ transient amplitude of FTG is increased. Also in contrast to most other experimental cardiomyopathies, (calcineurin overexpressing mice being an exception [17] in which the SR Ca^2+^ load decreases) (**Table S3**), the SR Ca^2+^ load of FTG increases, and, following excitation, generates a Ca^2+^ transient of increased amplitude. Since neither *I*_Ca,L_ amplitude nor inactivation under VC did differ between FTG and NTG, the source of the increased Ca^2+^ that likely augments the SR Ca^2+^ load in FTG compared to NFTG cannot be ascertained with certainty, particularly in the absence of action potential data in these two groups. Potential other sources of “extra” Ca^2+^ in FTG mice may be increased influx via NCX [1], [3], [4], [10] or efflux from mitochondria [18].

It is perplexing that the SR Ca^2+^ load increases during the evolution from NFTG to FTG, since *I*_Ca,L_ density does not increase, and Ca^2+^ efflux via NCX is augmented, it might be expected that mechanisms to reduce Ca^2+^ pumping into the SR might prevail: in many other HF models, SERCA2a becomes reduced, PLN increases, and its phosphorylation decreases [1], [7]. The increased SR Ca^2+^ load in FTG is apparently linked to an increased Ca^2+^ availability for SR pumping, as evidenced by an increase in diastolic Indo-1 fluorescence during pacing. The latter may also reflect an increase in the net cell Ca^2+^ load between the NFTG and FTG states.

Alterations in RyR [1] might also contribute to an increased RyR Ca^2+^ leak. Unlike some other heart failure models, RyR2 and RyR2-P decrease, in the FTG (c.f., **Table S3** **Online Supplement**), another conundrum. Some reports suggest that PKA phosphorylation of RyR2 has little functional relevance for diastolic Ca^2+^ release if SR Ca^2+^ levels remain constant [1], [2]. Marks and coworkers in many studies and perspectives, indict RyR2 and Ca^2+^ leak as causal of arrhythmias and sensitivities to heart failure [2], [19], [20]. There is a great deal of controversy surrounding the significance of both the increased Ca^2+^ leak theory, as well as the decreased SR Ca^2+^ content concept, as some studies have not been confirmed by others [1], [2], [21]. Our results, in fact, demonstrate that frank heart failure [10], [11] can occur in the context of **reduced** total RyR2, phosphorylated RyR2 at Serine 2809 and **increased** SR Ca^2+^ loading [22], [23]. This underscores the complexity of “heart failure”, and the caution that should be applied to any specific molecular conclusion at this point.

Some might argue that the FTG in which SR Ca^2+^ remains elevated does not represent a “good” model of heart failure. But what, then, is a “good model” of heart failure? A “good model” of heart failure should represent what happens in the human condition. The presently in vogue dictum is that a reduced SR Ca^2+^ load in end stage human HF is central to contractile dysfunction of human cardiomyocytes [10]. However, not all studies in human HF share this view [24]. In fresh, end stage failing hearts (a large number) obtained at transplant, using two different procedures, SR Ca^2+^ accumulation demonstrated slow uptake kinetics and a complete lack of Ca^2+^ release compared to non-failing samples, in which Ca^2+^ release was vigorous [25], [26]. These results, repeated on any human hearts are consistent with the present findings in the FTG myocytes. Further, a window of enhanced contractility, depending upon the stimulation rate, has been observed in human HF [1]. However, even in HF models that exhibit a reduced SR Ca^2+^ load, muscles or myocytes under study conditions that have been employed exhibit an increase in the diastolic Ca^2+^ at higher pacing rates *i.e.* similar patterns observed in the present study in FTG.

A recent study on failing human and mouse frank heart failure [27] has discovered a heretofore unrecognized gain of function change in the L-type Ca^2+^ channel. Channel availability and single channel activity were markedly altered, the culprit being an up regulation of the β_2_-subunit of the L-VDCC complex. Further, the ensemble single channel activities of the terminally failing mouse heart and human heart were identical. Thus, quantitative measurements of single L-type channel function are vital when considering mechanisms involved in frank heart failure in all models of HF, regardless of species.

Other possible mechanisms in the conundrum of the end stage HF phenotype include heart rate responses [10], diminished contractile performance [28], increased diastolic calcium [29]. Abnormal myofilament mechanisms [30] may explain, in part, the deficient myocardial function of the intact α_1C_TG, heart [4] but the mechanistic link between the two is not firm, because isolated cells, *e.g.*, those analyzed in the present study, have no preload or afterload, two factors that, not only regulate contraction, but also regulate myofilament Ca^2+^ binding and release characteristics. It is noteworthy, though, that regardless of the SR Ca^2+^ load, contractile failure in cells or muscles from end stage, dilated heart failure hearts is exacerbated with increasing pacing rates in virtually all studies employing different pacing rates [10], [11], [14]. Myofilament abnormalities other than Ca^2+^ binding or energetic fatigue, especially the activity of the β_2_-subunit of the L-VDCC, should continue to be recognized as mechanisms that underlie HF [10]. PKCα dephosphorylation of PLN was paralleled by diminished peak calcium transients.

Heart remodeling (dilatation), itself, leads to increased afterload, fibrosis, myofilament disarray and increased cell death [10], and each of these have been implicated in contractile dysfunction characteristic of FTG mice [4]. We observed that the ratio of TUNEL-positive cardiomyocytes to the total cardiomyocytes was higher in NFTG and FTG without signs of HF compared to littermate NTG (data not shown).

Of possible relevance to the α_1C_-TG model, overexpression of the β_2a_ subunit of the L-VDCC leads to enhanced *I*_Ca,L_ resulting in Ca^2+^ dependent apoptosis in adult myocytes [18], [31] consistent with recent findings [27]. Studies of the transition from hypertrophy to HF that occurs in SHR during advanced age [32] have also shown that increased cardiomyocyte apoptosis occurs [33].

To summarize, end stage cardiac failure phenotype is remarkably similar to that in other types of heart failure in which myocytes have a reduced SR Ca^2+^ load and reduced Ca^2+^ amplitude, at least under conditions that have been employed in these studies (**Table S1**). Similar heart failure phenotypes are associated with both an increased or decreased SR Ca^2+^ load as well as SR Ca^2+^ release following excitation. This suggests that there is a requirement for optimal Ca^2+^ transient amplitude to prevent HF, or that the mode of measurements, type of failure and how quickly measurements are made are of significance. Heterogeneity of Ca^2+^ release within and among cells in intact myocardium impairs both systolic and diastolic function and provokes arrhythmias [34]. Recent studies have indeed begun to address this issue in experimental heart failure models [2], [4], [7], [35], [36].

## Materials and Methods

FBV/n in-house bred 8–11 mo old α_1C_TG mice and their wild-type (NTG) littermates of the same age were used in all of the experiments. The animal protocols used in these studies were approved by the Animal Care Committees of each institution involved. At 9 mo of age some α_1C_TG mice begin to manifest a dilated cardiomyopathy with markedly impaired contractile function ***in vivo*** [4]. Upon sacrifice we observed α_1C_TG -mice at this age exhibited two phenotypes: the absence of gross cardiac dilatation and congestion, evidenced by the absence of pericardial and pleural effusions and atrial thrombi (n = 4), and gross cardiac dilatation with both pericardial and pleural effusions and left atrial thrombi (n = 4). We designated these two groups of mice at sacrifice as non-failing α_1C_TG (NFTG) and failing α_1C_TG (FTG), respectively, and structured our data analysis to compare some aspects of Ca^2+^ regulation and contraction characteristics of myocytes isolated from these hearts.

We measured Ca^2+^ transients in Indo-1-AM loaded myocytes at frequencies of stimulation ranging from 0.5–4 Hz. The same stimulation protocol was used to measure myocyte mechanics in both Indo-1-loaded and unloaded cells. The SR Ca^2+^ load was determined measuring caffeine induced Ca^2+^ transients in Indo-1-AM loaded cells. Inward Ca^2+^ current through the L-type channel was measured under step-voltage protocols. We carried out quantitative and traditional Western blotting for the Ca^2+^ handling proteins. FTG and NFTG subgroup comparisons were stratified prospectively and investigators were blinded when doing their experiments. All methods have been previously published and are detailed in the **Methods S1**.

### Statistics

Results are reported as mean ±SEM. Differences among NTG and NFTG and FTG cells were compared by ANOVA. A value of p<0.05 was considered significant.

## Supporting Information

Table S1 (0.13 MB DOC)

Table S2 (0.04 MB DOC)

Table S3 (0.05 MB DOC)

Methods S1 (0.06 MB DOC)
