# Supplementary material for: Dilated Cardiomyopathy with Increased SR Ca2+ Loading Preceded by a Hypercontractile State and Diastolic Failure in the α1CTG Mouse
Source: PLoS One. 2009 Jan 6;4(1):e4133. doi: 10.1371/journal.pone.0004133 (PMC2607013; doi:10.1371/journal.pone.0004133)
Supplement: Table S1 — (0.13 MB DOC) [file pone.0004133.s001.doc]

**Table S1. Genetically altered mouse models of heart failure**

| **Gene modified** | **Reference** | **Electrophysiological and calcium cycling changes** | **Phenotype** |
| --- | --- | --- | --- |
| L-type Ca^2+^ channel α_1_ subunit OE | Muth JN *et al.*[5]  Muth JN *et al.*[6]  Song LS *et al.*[2]  Bodi I *et al.* [7] | *I*_Ca_ and [Ca^2+^]_i_ increased in cardiomyocytes;  APD prolonged and Ito decreased in 8-10 month old mice | Hypertrophy and heart failure |
| Calsequestrin OE | Knollmann BC *et al.*[8]  Sato Y. *et al.*[9]  Jones LR *et al.*[10] | *I*_Ca_, *I*_to_, *I*_K1_ decreased, *I*_Ca_ inactivation slowed;  NCX increased by 33%;  APD prolonged, [Ca^2+^]_i_ and CICR decreased | Cardiac hypertrophy |
| Gαq OE | D’Angelo DD *et al.*[11]  Mitarai S *et al.*[12]  Dorn GW *et al.*[13]  Mende U *et al.*[14] | No change in *I*_Ca_; NCX, *I*_to_, *I*_K1_ decreased;  APD prolonged | Cardiac hypertrophy |
| Rab_1a_ GTPase OE | Wu G *et al.*[15] | *I*_Ca_ decreased; [Ca^2+^]_i_ amplitude did not change; time for 50% and 80% of maximal relaxation was depressed | Cardiac hypertrophy, progression to heart failure |
| Gαs OE | Kim SJ *et al.*[16]  Lader AS *et al.*[17] | *I*_Ca_ density decreased in adult mice;  [Ca^2+^]_i_ not changed, relaxation is shorter | Cardiomyopathy |
| K_v4.2_ DN OE | Wickenden AD *et al.*[18] | APD prolonged, *I*_to_, *I*_K1_ decreased;  [Ca^2+^]_i_ increased | Hypertrophy |
| Calcineurin OE | Molkentin JD *et al.*[19] Petrashevskaya NN *et al.*[20]  Dong D *et al.*[21]  Yatani A *et al.*[22]  Chu G *et al.*[23] | Increase in peak *I*_Ca_ density; faster *I*_Ca_ inactivation;  the density of *I*_to-f_, *I*_to-s_ and I_K-slow_ decreased;  APD prolonged [Ca^2+^]_i_ increased, enhanced SR function | Hypertrophy, cardiac arrhythmia  apoptosis |
| Triadin 1 OE | Kirchhefer U et al.[24] | [Ca^2+^]_i_ transient decayed at a slower rate; *I*_Ca_ was decreased; fast time inactivation constant of I_Ca_ was prolonged | Cardiac hypertrophy |
| PKCε OE | Takeishi Y *et al.*[25] | The amplitude of [Ca^2+^]_i_ transient was decreased; | Concentric cardiac hypertrophy |
| PKCβ_2_ OE | Wakasaki H *et al.*[26]  Takeishi Y et al.[27] | Reduction in myocyte contraction; amplitude of [Ca^2+^]_i_ transient did not change; cardiac troponin I may decrease myofilament Ca^2+^ responsiveness | Left ventricular hypertrophy |
| FKBP12 KO | Shou W *et al.* [28] | Defect in cardiac RYR2 function: increased open probability of the RYR2 channel, increased sensitivity to Ca^2+^-dependent activation; increased frequency and duration of Ca^2+^ sparks | Cardiomyopathy and heart failure |
|  | Xin HB *et al.* [29] | Neither the magnitude nor the kinetics of *I*_Ca_ was altered, peak [Ca^2+^]_i_ increased  Marked increase in amplitude and duration of Ca^2+^  sparks | Cardiac hypertrophy in male mice |
|  | Wehrens X *et al.*[30] | RyR2 channels exhibited altered single-channel properties including an increase in open probability under exercise-induced stimulation | Ventricular arrhythmia, sudden death during exercise |
| Junctin OE | Hong CS *et al.*[31] | *I*_Ca_ increased, APD increased in ventricular and atrial cells | Bi-atrial and bi-ventricular enlargment;  atrial fibrillation, bradycardia |
| CaMKIIδ_C_-OE | Maier LS *et al.*[32] | *I*_Ca_ was slightly increased, APD was prolonged, [Ca^2+^]_i_ transients and diastolic [Ca^2+^]_i_ reduced by 50%, enhanced Na^+^-Ca^2+^ exchange function | Dilated cardiomyopathy and heart  failure |
| CaMKIIδ_B_-OE | Zhang T *et al.*[33] | Decreased contractile function, increase in phosphatase activity | Cardiac hypertrophy |
| CaMKIV-OE | Passier R *et al.*[34]  Wu Y *et al.*[35] | Prolonged APD, reduced repolarizing K^+^ current  increased tendency for early afterdepolarization  triggering mechanism for arrhythmias  L-VDCC opening probability is increased | Slow progression of cardiac hypertrophy |
| Tropomyosin-OE | Muthuchamy M *et al.*[36] | Contraction and relaxation impaired *ex vivo* | Atrial enlargement, fibrosis |
| α_1_-Adrenoreceptor OE | Engelhardt S *et al.*[37]  Engelhardt S *et al.*[38] | Impaired [Ca^2+^]_i_ transient: time to 50% decline (T_50_) was increased by 81% | Progressive hypertrophy and heart failure |
| β_2_-Adrenoreceptor OE | Liggett SB *et al.*[39] | Reduced *I*_Ca_ density and Isoproterenol responsiveness | 350 times background βAR expression line developed dilated cardiomyopthy and died of heart failure |
