# Supplementary material for: Dilated Cardiomyopathy with Increased SR Ca2+ Loading Preceded by a Hypercontractile State and Diastolic Failure in the α1CTG Mouse
Source: PLoS One. 2009 Jan 6;4(1):e4133. doi: 10.1371/journal.pone.0004133 (PMC2607013; doi:10.1371/journal.pone.0004133)
Supplement: Table S2 — (0.04 MB DOC) [file pone.0004133.s002.doc]

**Table S2. Contraction Parameters at 0.5 Hz**

**A. Cells Loaded with Indo1-AM**

|  | NTG (n=12) | **FTG (n=14)** |
| --- | --- | --- |
| **Diastolic Length (μm)** | 120.8±4.2 | 116.4±6.7 |
| **Twitch Amplitude**  **(%/Diastolic Length)** | 3.19±0.58 | 3.03±0.4 |
| **Maximum Velocity of Shortening**  **(μm/sec)** | 62.94±10.2 | 61.4±6.9 |
| Maximum Velocity of Re-lengthening  **(μm/sec)** | 40.0±6.8 | 44.7±5.5 |

B. Cells Not Loaded with Indo1-AM

|  | NTG (n=20) | **FTG (n=23)** |
| --- | --- | --- |
| **Diastolic Length (μm)** | 116.4±4.2 | 117.0±3.6 |
| Twitch Amplitude  **(%/Diastolic Length)** | 5.26±0.46 | 6.9±0.4 *^#^ **^+^** |
| **Maximum Velocity of Shortening**  **(μm/sec)** | 105±9.9 | 134.3±7.9 *^#^ **^+^** |
| **Maximum Velocity of Re-lengthening**  **(μm/sec)** | 83.8±10.4 | 102.3±6.2 ***^+^** |

***ANOVA p<0.05**

**^#^p<0.05 vs. NTG**

**^+^p<0.05 vs. Indo 1-AM loaded FTG cells**

**p>0.05 for Indo loading and FTG interaction**
