# Supplementary material for: Dilated Cardiomyopathy with Increased SR Ca2+ Loading Preceded by a Hypercontractile State and Diastolic Failure in the α1CTG Mouse
Source: PLoS One. 2009 Jan 6;4(1):e4133. doi: 10.1371/journal.pone.0004133 (PMC2607013; doi:10.1371/journal.pone.0004133)
Supplement: Table S3 — (0.05 MB DOC) [file pone.0004133.s003.doc]

**Table S3. Evolution of changes in Ca^2+^ regulatory mechanisms in α_1C_TG mice with respect to wild type littermates (NTG)**





🡹

*I*_Ca,L_ Density

🡹

🡹

🡹

NCX Protein

🡻

🡹

ND

Ryanodine Receptor Phosphorylation PS-2809

🡻

🡻



Ryanodine Receptor Protein

🡻

🡻

🡹

PT-17

🡻

🡻

🡻

PS-16

Phospholamban

Phosphorylation

🡻

🡹



Phospholamban Protein

🡻

🡻



SERCA 2a

ND

ND

🡹

SR Ca^2+^ Release (Voltage Clamp)

🡹🡹

🡹

🡹

SR Ca^2+^ Release (AP)

🡹





SR Ca^2+^ Load (Caffeine)

8-11mo

**FTG**

8-11mo

**NFTG**

4mo

**α_1C_-TG [2]**
