# Supplementary material for: Dilated Cardiomyopathy with Increased SR Ca2+ Loading Preceded by a Hypercontractile State and Diastolic Failure in the α1CTG Mouse
Source: PLoS One. 2009 Jan 6;4(1):e4133. doi: 10.1371/journal.pone.0004133 (PMC2607013; doi:10.1371/journal.pone.0004133)
Supplement: Methods S1 — (0.06 MB DOC) [file pone.0004133.s004.doc]

**On line data Supplement**

**METHODS**

**Cardiomyocyte Isolation**

Single mouse cardiac myocytes were isolated from 8-11mo NTG and α_1C_TG mice as previously reported [1,2] with some modifications. Mice were anesthetized by Sodium Pentobarbital injection, and the heart was removed and cannulated under a dissecting microscope. The cannula was then placed on a perfusion apparatus and the heart perfused with nominally Ca^2+^ free solution (in mmol/L: NaCl 120, KCl 5.4, NaH_2_PO_4_ 1.2, Glucose 5.6, NaHCO_3_ 20, MgC1_2_ 1.2, Taurine 5, equilibrated to pH 7.3 by gassing with 95% 0_2_/5% CO_2_) for 3-4 min followed by same solution containing l mg/ml collagenase (Worthington, type II) and 50 μmol/L Ca^2+^ for 15-19 min. The ventricles were removed, cut into small pieces and gently aspirated with a plastic pipette to disperse the cells. The cells were centrifuged, collagenase solution removed and replaced with HEPES Solution (in mmol/L: NaCl 137, KCl 4.9, MgC1_2_ 1.2, NaH_2_PO_4_ 1.2, Glucose 15, HEPES 20 containing 125 μmol/L Ca^2+^). The myocytes were allowed to pellet via gravity and the supernatant was replaced with HEPES Solution containing 250 μmol/L Ca*^2+^*. This procedure was repeated with 500 μmol/L Ca^2+^ and the final suspension in 1 mmol/L of calcium.

**Contraction and Indo-1 Fluorescence Measurements**

Contraction and Indo-1 fluorescence measurements were made at room temperature in myocytes perfused with HEPES solution containing 1 mmol/L Ca^2+^. For ratiometric Ca^2+^ measurement, cells were loaded with 8 μmol/L indo-1/AM for 5-10 minutes, and cytosolic free Ca^2+^ concentration was indexed by the emission ratio of r_410/490_ [3]. Cell length was monitored by an optical edge tracking method [3]. Myocytes were field stimulated at 0.5, 1, 2, 3 and 4 Hz and measurements were obtained once steady-state contraction was achieved.

***I*_Ca_ Type Ca^2+^ Current Recordings**

L-type Ca^2+^ current was measured via the whole cell patch clamp technique using an Axon 200B amplifier. For selective examination of *I*_Ca_, various Na channel and K channel blockers were included in the perfusion solution and the pipette solution. The perfusion solution contains (in mmol/L): 0.02 tetrodotoxin, 10 CsCl, 4 4-AP, 137 NaCl, 1.2 MgcCl_2_, 10 HEPES, 2 CaCl_2_, 15 glucose. The pipette solution contains (in mmol/L): 100 CsCl, 10 NaCl, 20 TEA-Cl, 10 HEPES, 5 MgATP, 10 EGTA, 0.16 fluo 3-K. Both step-voltage clamp and ramp-voltage clamp protocols were used. During the step voltage clamp, *I*_Ca_ (inward calcium current) through the L-type voltage-dependent calcium channel is elicited by depolarizing voltage steps (380 ms) from -60 to +50 mV in 10 mV increments from a holding potential of -60 mV (Figure 2). During the ramp voltage clamp, the holding membrane potential was -80 mV. Following four 50-ms conditioning pulses to 0 mV at 950 ms interval, *I*_Ca_ was activated by a series of depolarization pulses from -80 mV to +60 mV than back to -80 mV with a series of voltage ramp (duration 50, 100, 200 and 500 ms) at 10 second interval (Figure 2B). The amplitude of *I*_Ca_ was measured as the difference between the peak inward current and the current at the end of the step- or ramp- clamp pulse. All of the experiments were performed at room temperature (23°C).

**Ca^2+^ Regulatory Protein Expression by Immunoblotting**

Cardiac homogenates were subjected to electrophoresis for the following Western Blots: SERCA 2a, RyR2, RyR2-P, PLN and PLN PS16 and PT17. Cardiac membrane fractions were used for the NCX Western Blot analysis. Preparation of cardiac homogenates was as follows: mouse ventricles were homogenized on ice in 1 ml of homogenization buffer (in mmol/L: 10 imidazole, 0.3 sucrose, 1 DDT, 1 sodium metabisulfite, 2 EDTA and 1 Complete Protease Inhibitor Cocktail tablet/25 ml of buffer (Roche)). Preparation of cardiac membrane fractions was as follows: Mouse ventricular tissue was homogenized in 1 ml of ice-cold lysis buffer (in mmol/L: 25 Tris, 5 EDTA, 5 EGTA, pH=7.4, one Complete Protease Inhibitor tablet/25 ml buffer (Roche)) and centrifuged at 800 g for 15 min at 4°C. The supernatant was then centrifuged at 100,000 g for 60 min at 4°C. The supernatant constitutes the cytosolic fraction. The pellet was resuspended in 500 μl of lysis buffer containing 0.5% TritonX-100 and centrifuged at 100,000 g for 60 min at 4°C. The resulting supernatant constitutes the soluble membrane fraction.

Quantitative Immunoblot Analysis for NTG and NFTG: Each sample was assayed in duplicates as in Harrer *et al*. [4]. Briefly, increasing concentrations of α_1C_TG and NTG cardiac proteins (1x, 2x and 4x) were subjected to electrophoresis in SDS polyacrylamide gels. For RyR2, 1x equals 15 μg of homogenate protein, 5 μg for PLN and 20 μg for SERCA2. Gels were transferred to membranes and these were blocked in TBS-5% and incubated with primary antibodies (1:5000 PLN, ABR MA3-922; 1:5000 PLN PS16, Cyclacel; 1:2500 PLN PT17, Cyclacel; 1:500 SERCA2, ABR MA3-919; 1:500 RyR, ABR MA3-916; 1:5000 RyR-P, Badrilla). Horseradish-peroxidase conjugated secondary antibodies and Enhanced Chemiluminescence reagents were used to visualize specific bands. Quantitation was done using densitometric analysis with Alpha Imager software. Densitometric values were plotted vs. μg of protein loaded and linear regression was performed to obtain the slopes. Slopes of NTG samples were compared to α_1C_TG, and are presented normalized to NTG values as means±SEM.

NCX immunoblot analysis: 80 μg (1x) of NTG and α_1C_TG membrane fractions were subjected to electrophoresis and transferred to nitrocellulose membranes. Membranes were treated as specified above (1:200 NCX, Swant). Quantitation was as indicated above. NCX protein levels relative to NTG are reported as means ± SEM.

The protocol mentioned above was also used for the quantitative Immunoblot Analysis for NTG and FTG. However, the protein concentration loaded was as follows: 50 μg for SERCA2a, NCX, RyR2 and RyR2-PS2809; 20 μg for PLN_t_, PLN_p_-PS16 and PLN_p_-PT17. Western-blotting was accomplished using the following antibodies: 1:500 PLN (ABR MA3-922); 1:5000 PLN_p_-PS16 (Badrilla); 1:5000 PLN_p_- PT17 (Badrilla); 1:1000 SERCA 2a (ABR MA3-919); 1:500 RyR2, (ABR MA3-916); 1:5000 RyR-PS2809 (Badrilla); 1:500 NCX (SWant). Activity of PKCα was determined from membrane to cytosol ratio using Western-blot (1:1000 PKCα antibodies from Santa Cruz). Cytoplasmic and membrane protein were isolated from ventricular tissue using Tris lysis buffer containing: (in mM) Tris-HCl 25 (pH=7.4), EGTA 5, EDTA 2, DTT 5, protease inhibitor cocktail tablets (Roche) and phosphatase inhibitors. 80 μg of protein homogenate was loaded per sample.

*Additional references for methods and Table 1*

1. Zhou YY, Wang SQ, Zhu WZ, Chruscinski A, Kobilka BK, et al. (2000) Culture and adenoviral infection of adult mouse cardiac myocytes: methods for cellular genetic physiology. Am J Physiol Heart Circ Physiol 279: H429-436.

2. Song LS, Guia A, Muth JN, Rubio M, Wang SQ, et al. (2002) Ca(2+) signaling in cardiac myocytes overexpressing the alpha(1) subunit of L-type Ca(2+) channel. Circ Res 90: 174-181.

3. Stern MD, Spurgeon HA, Hansford R, Lakatta EG, Capogrossi MC (1989) Optimum spectral windows to minimize quantum noise of ratiometric intracellular fluorescent probes. Cell Calcium 10: 527-534.

4. Harrer JM, Kiss E, Kranias EG (1995) Application of the immunoblot technique for quantitation of protein levels in cardiac homogenates. Biotechniques 18: 995-998.

5. Muth JN, Yamaguchi H, Mikala G, Grupp IL, Lewis W, et al. (1999) Cardiac-specific overexpression of the alpha(1) subunit of the L-type voltage-dependent Ca(2+) channel in transgenic mice. Loss of isoproterenol-induced contraction. J Biol Chem 274: 21503-21506.

6. Muth JN, Varadi G, Schwartz A (2001) Use of transgenic mice to study voltage-dependent Ca2+ channels. Trends Pharmacol Sci 22: 526-532.

7. Bodi I, Muth JN, Hahn HS, Petrashevskaya NN, Rubio M, et al. (2003) Electrical remodeling in hearts from a calcium-dependent mouse model of hypertrophy and failure: complex nature of K+ current changes and action potential duration. J Am Coll Cardiol 41: 1611-1622.

8. Knollmann BC, Knollmann-Ritschel BE, Weissman NJ, Jones LR, Morad M (2000) Remodelling of ionic currents in hypertrophied and failing hearts of transgenic mice overexpressing calsequestrin. J Physiol 525 Pt 2: 483-498.

9. Sato Y, Ferguson DG, Sako H, Dorn GW, 2nd, Kadambi VJ, et al. (1998) Cardiac-specific overexpression of mouse cardiac calsequestrin is associated with depressed cardiovascular function and hypertrophy in transgenic mice. J Biol Chem 273: 28470-28477.

10. Jones LR, Suzuki YJ, Wang W, Kobayashi YM, Ramesh V, et al. (1998) Regulation of Ca2+ signaling in transgenic mouse cardiac myocytes overexpressing calsequestrin. J Clin Invest 101: 1385-1393.

11. D'Angelo DD, Sakata Y, Lorenz JN, Boivin GP, Walsh RA, et al. (1997) Transgenic Galphaq overexpression induces cardiac contractile failure in mice. Proc Natl Acad Sci U S A 94: 8121-8126.

12. Mitarai S, Reed TD, Yatani A (2000) Changes in ionic currents and beta-adrenergic receptor signaling in hypertrophied myocytes overexpressing G alpha(q). Am J Physiol Heart Circ Physiol 279: H139-148.

13. Dorn GW, 2nd, Tepe NM, Wu G, Yatani A, Liggett SB (2000) Mechanisms of impaired beta-adrenergic receptor signaling in G(alphaq)-mediated cardiac hypertrophy and ventricular dysfunction. Mol Pharmacol 57: 278-287.

14. Mende U, Kagen A, Cohen A, Aramburu J, Schoen FJ, et al. (1998) Transient cardiac expression of constitutively active Galphaq leads to hypertrophy and dilated cardiomyopathy by calcineurin-dependent and independent pathways. Proc Natl Acad Sci U S A 95: 13893-13898.

15. Wu G, Yussman MG, Barrett TJ, Hahn HS, Osinska H, et al. (2001) Increased myocardial Rab GTPase expression: a consequence and cause of cardiomyopathy. Circ Res 89: 1130-1137.

16. Kim SJ, Yatani A, Vatner DE, Yamamoto S, Ishikawa Y, et al. (1999) Differential regulation of inotropy and lusitropy in overexpressed Gsalpha myocytes through cAMP and Ca2+ channel pathways. J Clin Invest 103: 1089-1097.

17. Lader AS, Xiao YF, Ishikawa Y, Cui Y, Vatner DE, et al. (1998) Cardiac Gsalpha overexpression enhances L-type calcium channels through an adenylyl cyclase independent pathway. Proc Natl Acad Sci U S A 95: 9669-9674.

18. Wickenden AD, Lee P, Sah R, Huang Q, Fishman GI, et al. (1999) Targeted expression of a dominant-negative K(v)4.2 K(+) channel subunit in the mouse heart. Circ Res 85: 1067-1076.

19. Molkentin JD, Lu JR, Antos CL, Markham B, Richardson J, et al. (1998) A calcineurin-dependent transcriptional pathway for cardiac hypertrophy. Cell 93: 215-228.

20. Petrashevskaya NN, Bodi I, Rubio M, Molkentin JD, Schwartz A (2002) Cardiac function and electrical remodeling of the calcineurin-overexpressed transgenic mouse. Cardiovasc Res 54: 117-132.

21. Dong D, Duan Y, Guo J, Roach DE, Swirp SL, et al. (2003) Overexpression of calcineurin in mouse causes sudden cardiac death associated with decreased density of K+ channels. Cardiovasc Res 57: 320-332.

22. Yatani A, Honda R, Tymitz KM, Lalli MJ, Molkentin JD (2001) Enhanced Ca2+ channel currents in cardiac hypertrophy induced by activation of calcineurin-dependent pathway. J Mol Cell Cardiol 33: 249-259.

23. Chu G, Carr AN, Young KB, Lester JW, Yatani A, et al. (2002) Enhanced myocyte contractility and Ca2+ handling in a calcineurin transgenic model of heart failure. Cardiovasc Res 54: 105-116.

24. Kirchhefer U, Neumann J, Baba HA, Begrow F, Kobayashi YM, et al. (2001) Cardiac hypertrophy and impaired relaxation in transgenic mice overexpressing triadin 1. J Biol Chem 276: 4142-4149.

25. Takeishi Y, Ping P, Bolli R, Kirkpatrick DL, Hoit BD, et al. (2000) Transgenic overexpression of constitutively active protein kinase C epsilon causes concentric cardiac hypertrophy. Circ Res 86: 1218-1223.

26. Wakasaki H, Koya D, Schoen FJ, Jirousek MR, Ways DK, et al. (1997) Targeted overexpression of protein kinase C beta2 isoform in myocardium causes cardiomyopathy. Proc Natl Acad Sci U S A 94: 9320-9325.

27. Takeishi Y, Chu G, Kirkpatrick DM, Li Z, Wakasaki H, et al. (1998) In vivo phosphorylation of cardiac troponin I by protein kinase Cbeta2 decreases cardiomyocyte calcium responsiveness and contractility in transgenic mouse hearts. J Clin Invest 102: 72-78.

28. Shou W, Aghdasi B, Armstrong DL, Guo Q, Bao S, et al. (1998) Cardiac defects and altered ryanodine receptor function in mice lacking FKBP12. Nature 391: 489-492.

29. Xin HB, Senbonmatsu T, Cheng DS, Wang YX, Copello JA, et al. (2002) Oestrogen protects FKBP12.6 null mice from cardiac hypertrophy. Nature 416: 334-338.

30. Wehrens XH, Lehnart SE, Huang F, Vest JA, Reiken SR, et al. (2003) FKBP12.6 deficiency and defective calcium release channel (ryanodine receptor) function linked to exercise-induced sudden cardiac death. Cell 113: 829-840.

31. Hong CS, Cho MC, Kwak YG, Song CH, Lee YH, et al. (2002) Cardiac remodeling and atrial fibrillation in transgenic mice overexpressing junctin. Faseb J 16: 1310-1312.

32. Maier LS, Zhang T, Chen L, DeSantiago J, Brown JH, et al. (2003) Transgenic CaMKIIdeltaC overexpression uniquely alters cardiac myocyte Ca2+ handling: reduced SR Ca2+ load and activated SR Ca2+ release. Circ Res 92: 904-911.

33. Zhang T, Johnson EN, Gu Y, Morissette MR, Sah VP, et al. (2002) The cardiac-specific nuclear delta(B) isoform of Ca2+/calmodulin-dependent protein kinase II induces hypertrophy and dilated cardiomyopathy associated with increased protein phosphatase 2A activity. J Biol Chem 277: 1261-1267.

34. Passier R, Zeng H, Frey N, Naya FJ, Nicol RL, et al. (2000) CaM kinase signaling induces cardiac hypertrophy and activates the MEF2 transcription factor in vivo. J Clin Invest 105: 1395-1406.

35. Wu Y, Temple J, Zhang R, Dzhura I, Zhang W, et al. (2002) Calmodulin kinase II and arrhythmias in a mouse model of cardiac hypertrophy. Circulation 106: 1288-1293.

36. Muthuchamy M, Boivin GP, Grupp IL, Wieczorek DF (1998) Beta-tropomyosin overexpression induces severe cardiac abnormalities. J Mol Cell Cardiol 30: 1545-1557.

37. Engelhardt S, Hein L, Wiesmann F, Lohse MJ (1999) Progressive hypertrophy and heart failure in beta1-adrenergic receptor transgenic mice. Proc Natl Acad Sci U S A 96: 7059-7064.

38. Engelhardt S, Boknik P, Keller U, Neumann J, Lohse MJ, et al. (2001) Early impairment of calcium handling and altered expression of junctin in hearts of mice overexpressing the beta1-adrenergic receptor. Faseb J 15: 2718-2720.

39. Liggett SB, Tepe NM, Lorenz JN, Canning AM, Jantz TD, et al. (2000) Early and delayed consequences of beta(2)-adrenergic receptor overexpression in mouse hearts: critical role for expression level. Circulation 101: 1707-1714.
